# Age, growth, and energy storage of the subterranean fish *Triplophysa rosa* (Cypriniformes: Nemacheilidae) from Chongqing, China

**DOI:** 10.1186/s12862-023-02186-y

**Published:** 2023-12-07

**Authors:** Yuan Xu, Yangyang Jing, Jing Zhou, Rui Long, Juanzhu Meng, Ya Yang, Yiping Luo

**Affiliations:** 1 Key Laboratory of Freshwater Fish Reproduction and Development, Ministry of Education, School of Life Sciences, Southwest University, Chongqing, 400715 China; 2 Department of Clinical Medicine, Chongqing Medical and Pharmaceutical College, Chongqing, 401331 China

**Keywords:** Cavefish, Growth, Storage, *Triplophysa rosa*, VBGF

## Abstract

**Background:**

This study explores the age, growth, and energy storage of *Triplophysa rosa*, a troglobitic cavefish. A total of 102 wild *T. rosa* specimens were collected in Wulong County, Chongqing, China, between 2018 and 2022, with otoliths used for age determination.

**Results:**

The earliest mature individuals were determined to be 4.8 years old, while the maximum ages for females and males were estimated at 15.8 years and 12.2 years, respectively. The length (*L*, cm)-weight (*W*, g) relationship was found to be the same for both sexes, following the eq. *W* = 0.0046 *L*^3.03^. Von Bertalanffy growth models were applied to the total length-at-age data, resulting in an asymptotic length of 23.4 cm and a *K*-parameter of 0.060 year^−1^. The body content of protein, ash, and glycogen did not show a significant correlation with the total length of *T. rosa*. However, both lipid and energy content exhibited a significant increase with total length. The lipid content ranged from 40.5 to 167.1 mg g^−1^, while the energy content ranged from 4.50 to 11.39 kJ g^−1^, indicating high storage features of *T. rosa*.

**Conclusions:**

The results affirm that *T. rosa* exhibits life traits conducive to its population dynamics in cave conditions, characterized by slow growth, small size, and high lipid energy storage.

## Introduction

Cavefish, which inhabit subterranean river environments, encompass three distinct types: troglobites, which are exclusive to subterranean habitats; troglophiles, capable of residing in either hypogean or epigean habitats throughout their entire lifespan; and trogloxenes, which spend only a part of their life cycle in the subterranean habitat [1]. Among these types, troglobites exhibit remarkable adaptations to subterranean life, characterized by specialized traits such as degenerated eyes and reduced surface pigmentation, making them particularly intriguing subjects for the study of evolution in extreme environments with limited resources and absence of light [1–3]. Extensive research has delved into various aspects including population ecology, genetics, development, physiology, behavior, and evolution of troglobitic cavefish [1, 2, 4–13].

Age and growth represent critical components of the life history of fish, influencing species’ energy acquisition and allocation strategies while being intricately linked to selective pressure and environmental adaptation [14–17]. Environmental variables, particularly food availability, play a pivotal role in energy intake and allocation among essential processes such as maintenance, growth, storage, and reproduction, ultimately shaping species’ fitness [18–22]. It is well-documented that fish exhibit slow growth in environments with limited food resources and tend to allocate a significant portion of their energy to body fat storage [23]. Moreover, predation pressure, in addition to food sources, influences the growth patterns of animals [24, 25]. Higher predation pressure often leads to accelerated growth rates of fish as larger body sizes serve to minimize the risk of predation [26–29]. Therefore, the absence of primary producers and predators in cave aquatic habitats imposes restrictions on food resources and predation pressure, culminating in the assumption that troglobitic cavefish display slow growth rates and significant body lipid and energy storage [10, 30]. Notably, the traits of slow growth and small size have been observed in several species of troglobitic cavefish, including *Typhlichthys subterraneus*, *Amblyopsis spelaea*, *Amblyopsis rosae*, *Astyanax mexicanus*, and *Ituglanis passensis* [11, 31, 32].

The karst habitats of Southwest China harbor a substantial diversity of cavefish, with 148 identified species found in this region across four families (Cyprinidae, Cobitidae, Nemacheilidae, and Amblycipitidae), including 78 troglomorphic species [33]. Among these, *Triplophysa rosa* stands out as a troglobitic cavefish exclusively inhabiting the groundwater accessible from Mengchongtang Cave in Huolu Town, Wulong County, Chongqing, China [34, 35]. *T. rosa* is an omnivorous species known to feed on *Caridina serratirostris* and a variety of plants [36], and it boasts troglomorphic and cave-specialized physiological characteristics, including a colorless body, degraded eyes, and notably low metabolic rate [34–38]. Recent research has further unveiled its low rate of molecular evolution, relaxed purifying selection, and lack of a behavioral stress response [12, 13]. However, despite the significance of understanding how natural selection and evolutionary forces shape organisms to adapt to environmental challenges [39, 40], investigations into fitness-related life history traits in this species have been lacking. To glean insights into the life-history strategies of cavefish, this study undertook an examination of the age, growth, and body energy storage of *T. rosa*. Data regarding the body length and age of *T. rosa* were leveraged to estimate the traditional von Bertalanffy growth function (VBGF) [41], offering a comparative analysis with various species of epigean fish.

## Results

### Length-weight relationship and isometric growth

**Fig. 1:**
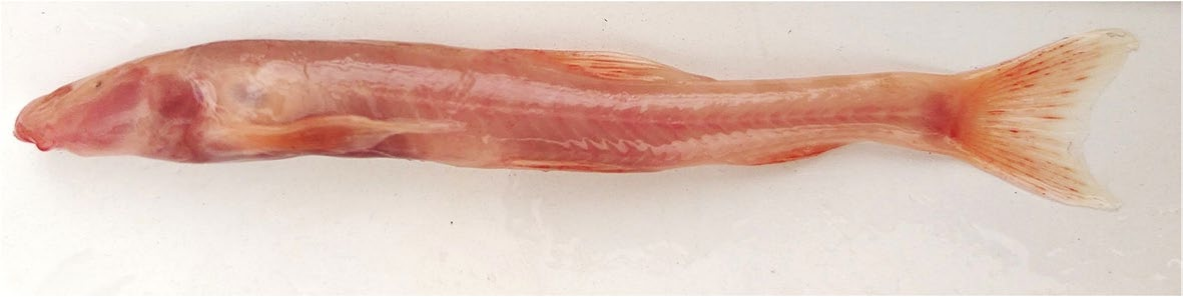
The photo of an individual of *Triplophysa rosa*

**Fig. 2:**
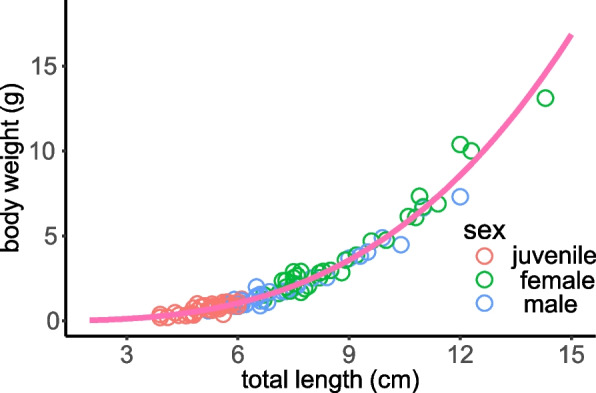
The length and weight relationships of *Triplophysa rosa*

A total of 102 wild *T. rosa* specimens were collected in Wulong County, Chongqing, China, exhibiting a wide range in total length (3.9 to 14.3 cm) and body weight (0.15 to 13.12 g) (Fig. 1). The earliest maturing individual was observed to be 4.8 years old, thus individuals below this age were categorized as juveniles for modeling purposes. Among the samples, 32 were identified as females (aged 4.8 to 15.8 years, body weight range: 1.25 to 13.12 g, total length range: 6.7 to 14.3 cm), 33 were males (aged 4.8 to 12.2 years, body weight range: 0.60 to 7.31 g, total length range: 5.2 to 12.0 cm), and 37 were juveniles (aged 3.3–5.5 years, body weight range: 0.15 to 1.28 g, total length range: 3.9 to 6.1 cm). The analysis revealed no significant difference in the length-weight relationship between females and males (intercept: *t* = 0.284, *P* = 0.778; slope: *t* = 0.081, *P* = 0.936). Consequently, a unified length-weight relationship was established for all samples of *T. rosa*: *W* = 0.0046 *L*^3.03^ (*R*^2^ = 0.945, *P* < 0.001), with the exponent not differing significantly from 3 (*t* = 0.397, *P* = 0.692) (Fig. 2).

### Precision of age estimation using otoliths

**Fig. 3:**
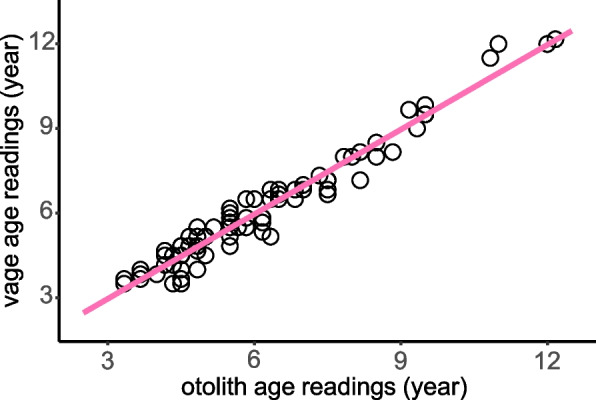
The correlation of age reads from otoliths and vertebrae of *Triplophysa rosa*

The study found a significant correlation between otoliths and vertebrae measurements in *T. rosa* (vertebrae = otoliths-0.035, *R*^2^ = 0.953, *P* < 0.001) (Fig. 3). Notably, for older individuals, otoliths exhibited superior between-reading precision for age estimation compared to vertebrae. In the case of the oldest individual, only the otolith reading provided clear data. Otoliths displayed ACV (Average Coefficient of Variation) values of 5.19% and APE (Average Percent Error) values of 3.89%, both lower than those of vertebrae (ACV: 6.4%; APE: 4.8%). Consequently, the age readings derived from the otoliths were utilized to model the growth function of *T. rosa*.

### Slow growth and small body size are shared traits of *Triplophysa*

**Fig. 4:**
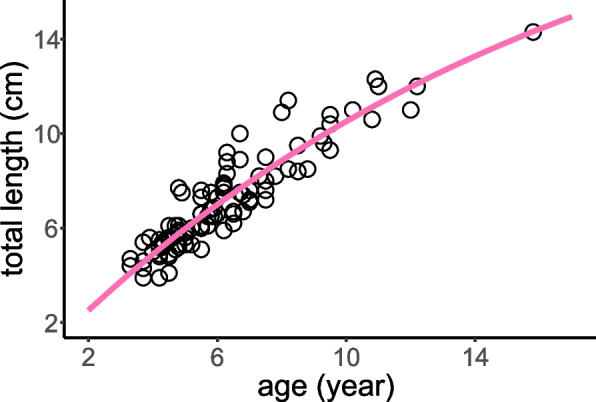
The von Bertalanffy growth curves of *Triplophysa rosa*

**Fig. 5:**
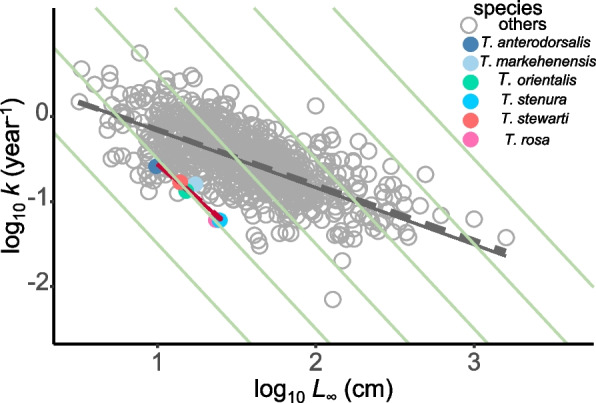
Auximetric plot comparing the von Bertalanffy growth parameters among fishes Species other than *Triplophysa* were extracted from FishBase (July 2022), and the von Bertalanffy growth parameters of *Triplophysa* other than *Triplophysa rosa* were quoted from the literature [42–45, 47]. Every parameter was transformed by a log10 factor. *Triplophysa* fish are shown by colored circles, whereas other species are represented by gray circles. On the link between *k* and *L*_*∞*_, both linear models (dashed line) and phylogenetic generalized linear models (solid line) were used. The gray lines show relationships among other species, while the red lines show relationships among *Triplophysa* species. The green lines connect the *Φ*’ values, the direct indication of growth performance

Among the five data series, only the overall data and the data combined by juveniles and females yielded statistically significant growth parameters for VBGF modeling (Table 1, Fig. 4), exhibiting comparable values of both *L*_∞_ and *K*. To facilitate comparison with other fish species, the growth parameters of VBGF modeled using the total data were adopted (Table 2, Fig. 5). The estimates of *L*_∞_ (23.4 cm), *K* (0.060 year^−1^), and *Φ’* (1.52) for *T. rosa* fell within the range observed in other species of *Triplophysa* (*K*: 0.060–0.26; *L*_*∞*_: 9.8–24.7; *Φ’*: 1.40–1.68). Moreover, the values of *K* (*t* = 3.768, *P* = 0.006), *L*_*∞*_ (*t* = 5.230, *P* = 0.001), and *Φ’* (*t* = 23.8, *P* < 0.001) for all *Triplophysa* were notably smaller than those of other fish species. Further analysis revealed a negative correlation between the *L*_∞_ value and *K* value across the six species of *Triplophysa*, as evidenced by the equation log_10_ *K* = − 1.64*log_10_ *L*_∞_ + 1.09 (*R*^2^ = 0.91, *P* = 0.003). Controlling for phylogenetic effects via phylogenetic generalized linear models (PGLS) yielded a very similar correlation: log *K* = − 1.63*log_10_ *L*_∞_ + 1.07 (*R*^2^ = 0.91, *P* = 0.013).

**Table 1:** The von Bertalanffy growth parameters of *Triplophysa rosa*

Data	*L*_*∞*_			*K*			*t*_0_		
	Mean±SE	*t*	*P*	Mean±SE	*t*	*P*	Mean±SE	*t*	*P*
Total	23.4±6.5	3.604	<0.001	0.060±0.026	2.313	0.023	0.12±0.60	0.196	0.845
female	102.17±754.1	0.135	0.893	0.008±0.065	0.122	0.904	-3.90±5.54	-0.704	0.487
male	271.7±433.9	0.051	0.960	0.003±0.061	0.50	0.961	-2.31±3.19	-0.725	0.474
female&juvenile	21.1±4.99	4.235	<0.001	0.077±0.030	2.533	0.014	0.60±0.54	1.096	0.277
male&juvenile	55.1±86.9	0.634	0.528	0.018±0.033	0.546	0.587	-1.16±1.02	-1.142	0.258

Parameters include asymptotic length (*L*_*∞*_, cm), growth rate (*K*, year^−1^), and the theoretical age at a length of zero (*t*_0_, year)

**Table 2:** The von Bertalanffy growth parameters comparison among species of *Triplophysa*

Species	Habitat type	*L*_*∞*_ (cm)	*K* (year^-1^)	*t*_*0*_ (year)	*Φ’*	Temperature (^o^C)	Altitude (m)	References
*T. rosa*	troglobitic fish	23.4	0.060	0.12	1.52	15	350	The present study
*T. anterodorsalis*	epigean fish	9.8	0.26	-2.09	1.40	17	1600	Wang & Liang 2017 [42]
*T. markehenensis*	epigean fish	17.3	0.16	-0.53	1.68	4	3600	Zhang et al. 2010 [43]
*T. stenura*	epigean fish	24.7	0.06	0.17	1.56	3	4200	Deng et al. 2010 [44]
*T. orientalis*	epigean fish	15.2	0.13	0.02	1.49	7	3500	Li et al. 2016 [45]
*T. stewarti*	epigean fish	13.9	0.17	2.10	1.51	4	4600	Tian et al. 2022 [46]

Parameters include asymptotic length (*L*_*∞*_, cm), growth rate (*K*, year^−1^), the theoretical age at a length of zero (*t*_0_, year), growth performance index (*Φ*’), the temperature and altitude of the species’ habits

### High lipid and energy content of *T. rosa*

**Fig. 6:**
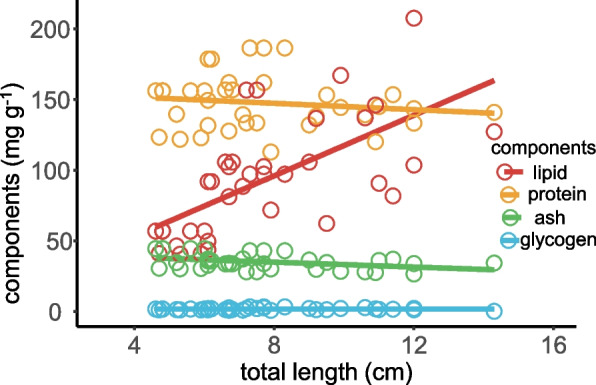
Variations in the body compositions with total length of *Triplophysa rosa*

**Fig. 7:**
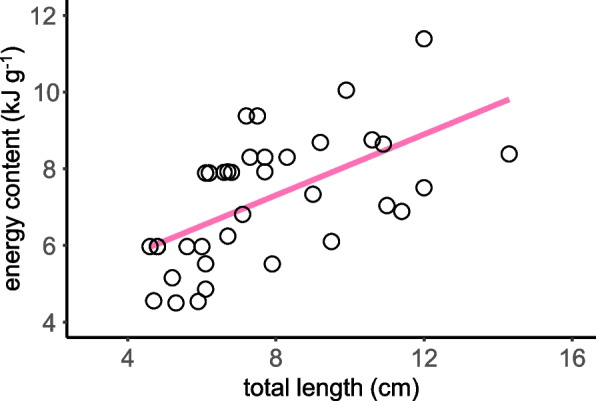
Variations in energy content with total length of *Triplophysa rosa*

A consistent but moderate decline was observed in the contents of protein, ash, and glycogen with increasing total length (Fig. 6). However, no significant relationship was identified between these variables and body length. In contrast, the lipid content ranged from 40.5 to 167.1 mg g^−1^ and exhibited a significant increase with the total length of *T. rosa*, characterized by a slope of 11.3 (*R*^2^ = 0.453, *P* < 0.001), which differed significantly from the slopes of protein (*t* = − 8.461, *P* < 0.001), ash (*t* = − 10.070, *P* < 0.001), and glycogen (*t* = − 15.567, *P* < 0.001). Meanwhile, the energy content ranged from 4.50 to 11.39 kJ g^−1^ and displayed a positive correlation with the total length, characterized by a slope of 0.397 (*R*^2^ = 0.341, *P* < 0.001) (Fig. 7).

## Discussion

Our findings demonstrate that the troglobitic fish *T. rosa* exhibits slow growth compared to the majority of epigean fish. Previous studies have suggested that the slow growth of troglobitic cavefish is likely attributed to the scarcity of food resources in the cave environment [48, 49]. Additionally, the absence of predators in cave environments diminishes the advantage of fast growth [25, 50, 51]. Interspecific analyses have indicated a positive correlation between growth performance and the metabolic rate of fish [47, 52]. Consistent with the characteristics of troglobites, *T. rosa* displays a notably low metabolic rate (38.3 mg O_2_ kg^−1^ h^−1^ at 15 °C) compared to the majority of epigean fish species [38].

The evolution of life history traits is influenced by environmental conditions [47, 52]. The slow growth of *T. rosa* may be a consequence of cave habitat selection or a contributing factor to its successful adaptation to the cave environment. When comparing the growth rate, asymptotic length, and growth performance index of *T. rosa* with the five reported *Triplophysa* species (all epigean fish, Table 2), we found that the measures of *T. rosa* fell within the range of those closely related epigean fish [42–46]. Furthermore, compared to 734 species from FishBase (July 2022), *Triplophysa* species exhibit lower growth rates, asymptotic lengths, and growth performance indices, as indicated in an auximetric plot [15, 53] (Fig. 5). This suggests that, rather than being unique to the troglobitic cavefish *T. rosa*, slow growth and small body size are shared traits among all studied *Triplophysa*. These traits of slow growth and small body size may represent a form of preadaptation to the cave habitat [1], enabling many *Triplophysa*, species, including *T. rosa*, to thrive in cave environments with minimal structural change [54, 55].

As indicated by prior research, the origin and evolution of the *Triplophysa* genus are closely tied to the Tibetan Plateau uplift, with only an 18-million-year since the divergence of the epigean river-dwelling lineage and the karst cave lineage [56–59]. Recent comparative genomic studies have revealed that the troglobitic cavefish *T. rosa* and the epigean fish *T. tibetana* diverged 25.5 million years ago [12], suggesting that *T. rosa* may not have specifically evolved for cave adaptation. Instead, its ability to thrive in cave conditions is likely a result of its preexisting life history traits, such as slow growth and small body size. Similarly, preadaptation to cave environments has been proposed in other fishes, such as the scotophilic behaviors observed in surface-living populations of *Astyanax mexicanus* and the cave-dwelling capabilities of epigean-eyed species in the *Sinocyclocheilus* genus [60, 61].

The youngest mature individuals of the troglobitic cavefish *T. rosa* were found to be 4.8 years old, significantly older than the reported maturity ages of epigean fish species, such as *T. bleekeri*, *T. yarkandensis*, *T. stoliczkae*, and *T. stewarti*, which typically mature at 1–2 years old [46, 62–64]. This suggests that *T. rosa* is a late-maturing *Triplophysa* species. The protracted generation period resulting from late maturity leads to a low mutation rate, consequently contributing to a slower evolution rate [65–67]. It has been suggested that *T. rosa*’s genome exhibits slower molecular evolution and lower mutation rates compared to closely related epigean fish species [12].

The exponent values of the length-weight relationships of *T. rosa* (3.03) indicate isometric growth, signifying that fish grow at a consistent rate in terms of their bone and muscle weights [68]. Both the ash content and the protein content of *T. rosa* demonstrated a concurrent pattern with increasing body size. However, the energy contents of *T. rosa* increased with total length (Fig. 7), attributed to the increased lipid content, suggesting an increase in energy storage as the body grows. The highest measured lipid content in *T. rosa* was 167.1 mg g^−1^, surpassing the range used to classify high-lipid fish, which typically ranges from 85 mg g^−1^ to 153 mg g^−1^ [69]. Variations in lipid concentration with growth may reflect the energy trade-off between growth and storage during the life history of fish [27–29]. In instances of limited food resources, animals cannot simultaneously meet the energy requirements for growth and storage, leading to a trade-off [17, 40]. To overcome the scarcity of environmental food resources, troglobitic cavefish must maximize energy storage. The findings suggest that high lipid and energy content may be a specialization of *T. rosa* for cave settings. According to a recent study, genes involved in lipid metabolism in *T. rosa* underwent relaxed purifying selection [12]. Research on other troglobitic cavefish has also observed lipid deposition-related features, such as increased visceral adipose tissue, larger visceral adipocytes, and high expression of lipogenesis genes in cave-dwelling *Astyanax mexicanus* [70–72], as well as the distinctive humpback shape for energy storage in *Sinocyclocheilus* troglobitic cavefishes [73]. This indicates that lipid deposition may be a common characteristic of troglobitic cavefish.

## Conclusions

Our study highlights the slow growth, small size, and high lipid energy storage exhibited by *T. rosa*. These traits could be preadaptation that existed before the species colonization of caves, rather than adaptations developed after entering this environment. This insight into the evolutionary traits of cave-dwelling species not only advances our understanding of their unique characteristics but also has implications for the study of adaptation and speciation in diverse ecological niches.

## Methods

### Sample collection and age identification

This study focused on *T. rosa* specimens found exclusively in the subterranean waters of Mengchongtang Cave [34, 35], located at coordinates N 29.397489, E 107.915402, Alt 235.2 m in Huolu Town, Wulong County, Chongqing. The cave is situated in a region experiencing a subtropical monsoon climate, characterized by an average annual temperature ranging between 14 and 24 °C and a vegetation coverage of over 65%. Comprised of carbonate rocks, the cave is situated in the well-developed karst zone of Southwest China [74]. While the geological and hydrological characteristics within the cave remain poorly understood, only two fish species, *T. rosa*, and a newly recorded species, *T. wulongensis*, have been identified within its confines [75].

The collection of fish specimens occurred biannually in May and October from 2020 to 2021, coinciding with the periods when *T. rosa* is known to be accessible in the cave’s water. The total sample size comprised 102 individuals, with 15 collected in May and 31 in October of 2020, and 49 in May and 7 in October of 2021. All procedures related to the collection and handling of fish samples were conducted following the guidelines and regulations stipulated by the Institutional Animal Care and Use Committee of Southwest University, Chongqing, China (IACUC-20210119-01) and the local fisheries management authorities. Following their collection, the living fish were transported by vehicle to the fish laboratory at Southwest University, situated 245 km from the collection site. Subsequently, the fish was anesthetized using MS-222 (0.15 g L^−1^) for approximately 5 minutes. The total length was measured using a caliper at 0.1 cm precision, and body weight was 0.01 g using an electronic scale, respectively, prior to their dissection. The gonads were utilized to distinguish between females, males, and juveniles, and for each specimen, three portions of the posterior vertebrae and two lapillus otoliths were retrieved. These otoliths and vertebrae were carefully cleaned, placed into centrifuge tubes, and stored at − 20 °C. The entire body of the fish was packed in plastic bags and frozen for subsequent analysis of its raw chemical components. To ensure the accurate age determination, both the vertebrae and lapillus otoliths of *T. rosa* were utilized. A pair of opaque/hyaline bands, which form annually and are described by Marriott et al. [76], was used to determine the age of each fish.

On the micro slide, the lapillus otolith sample was embedded in neutral resin and then polished using high mesh sandpaper (3000–10,000 mesh) until the otolith primordium became visible. Subsequently, xylene was employed to render the otolith sample translucent, and images were captured using a microscope (EV5680B, Aigo Digital Technology CO., Ltd., Beijing, China), while adjustments to the transmitted and reflected light were made to enhance the visibility of the annual ring.

The vertebrae sample underwent treatment with 1% NaOH solution in a boiling water bath to remove any connected muscle fibers and other materials. Following this, the vertebrae sample was immersed in ethanol to remove any residual grease. Each vertebra was then carefully cut longitudinally and placed vertically on the microslide. The longitudinal section was meticulously polished with 3000 mesh sandpaper to reveal the birthmark and edge rings [77]. Subsequently, annual rings were observed under a microscope after the application of a few drops of xylene.

The annual rings of otoliths (*t*_ot_, year) and vertebrae (*t*_ve_, year) were independently counted three times by a single observer, with readings conducted once monthly. In instances where variations in age counts arose from the three repeats, the samples were re-evaluated to establish a consensus final age. The average ACV and APE were used to assess the accuracy of the age readings for the vertebrae and otoliths [78].

### Growth modeling

The relationship between the total length (*L*, cm) and body weight (*W*, g) of *T. rosa* was expressed by the equation [79]: *W* = *aL*^*b*^, where *a* is a coefficient and *b* is an exponent. These parameters were estimated using the least square method applied to plots of log (*L*) versus log (*W*). The growth pattern of *T. rosa* was assessed using the classical VBGF [41]:

$$L={L}_{\infty}\left(1-{e}^{-K\ \left(t-t0\right)}\right)$$

where *L* denotes the total length (cm), *L*_∞_ stands for the asymptotic length (cm), *K* represents the rate at which *L*_∞_ is approached (year^−1^) and is commonly known as the growth rate, *t* is the age (year), and *t*_0_ signifies the theoretical age at a length of zero (year). In line with the approach of Pauly and Munro [80], an auximetric plot was used to compare the VBGF parameters *L*_*∞*_ and *K* using the index of growth performance *Φ*’, defined as:

$${\varPhi}^{\prime }=\log K+2\log\ {L}_{\infty }$$

To contextualize the VBGF parameters of *T. rosa*, data from 734 fish species were retrieved from FishBase in July 2022 [81], supplemented with information from five closely related epigean fish species of the genus *Triplophysa* obtained from published works [42–46].

### Body crude chemical composition

To determine the dry weight (g) and water weight (g), the stomach contents were removed and the fish body was dried to a consistent weight at 70 °C in an electrothermostatic blast oven (DHG-9070A, Qixin, Shanghai). Subsequently, the dried fish sample was pulverized into a powder, and the protein content (mg g^−1^) was analyzed using the Kjeldahl technique. The lipid content (mg g^−1^) was determined using the Soxhlet extraction method with ether as the extraction solvent, while the glycogen content (mg g^−1^) was determined using the anthrone technique. Additionally, the ash content (mg g^−1^) was measured through muffle furnace incineration at 550 °C for 7 hours. The energy content (E, kJ g^−1^) was calculated using the values of 23.6 kJ g^−1^ for protein, 39.5 kJ g^−1^ for fat, and 17.2 kJ g^−1^ for glycogen [82, 83]. To ensure sufficient sample size for chemical analysis, small individuals of comparable size (body length difference within 2 mm) were pooled and the mean values of body length, body mass, and chemical composition within each pool constituted a single sample.

### Statistical methods

Experimental data were analyzed using Excel 2010 (Microsoft Corporate, Redmond, WA, USA) and R. The values of total length and body weight were log_10_-transformed to analyze their correlation by fitting linear models. The sexual difference of the regression slopes was tested using sex as a covariate, and a *t-*test was used to compare the exponent of the length-weight relationship with 3, the slope of an isometric growth function. Additionally, the correlation between otolith readings and vertebra readings was analyzed through fitting linear models. The VBGF was fitted using nonlinear least squares with five series of data, including the data of all fish, the sex-specific data of females and males, and the mixed data of juveniles and females as well as juveniles and males. The interspecific relationship between the growth coefficient (*K*) and the asymptotic length (*L*_∞_) was analyzed using PGLS in the “caper” package, with log-transformed *K* and *L*_∞_ before analysis. The fish phylogeny was obtained from the “fishtree” package [84], and the branch length transformations were optimized using the maximum likelihood method [85]. The relationships between body composition and total length were fitted by linear models. Data were plotted using the ggplot2 package [86]. Differences were considered significant when the *P* value was less than 0.05.

## Data Availability

The datasets supporting the conclusions of this article are available in the figshare repository, 10.6084/m9.figshare.24190221.
